# Evaluation of the Feed Nutritional Value of Noni (*Morinda citrifolia*) Meal for Holstein Dairy Cows

**DOI:** 10.3390/ani12172196

**Published:** 2022-08-26

**Authors:** Sang-Hoon Keum, Won-Seob Kim, Jalil Ghassemi Nejad, Jae-Sung Lee, Yong-Ho Jo, Ki-Yeon Park, Yoo-Rae Kim, Jang-Hoon Jo, Hong-Gu Lee

**Affiliations:** 1 Department of Animal Science, Konkuk University, Seoul 05029, Korea; 2 Department of Animal Science, Michigan State University, East Lansing, MI 48824, USA

**Keywords:** noni meal, Holstein dairy cow, feed-nutritional value, milk fatty acids

## Abstract

**Simple Summary:**

Three follow-up experiments as one set were conducted to evaluate the feed nutritional value of noni (*Morinda citrifolia*) meal to be included in the diet of Holstein dairy cows. An in vitro study was conducted to explore the effect of noni meal on rumen fermentation characteristics. An in situ study was conducted to evaluate the rumen degradation characteristics of noni meal, using wheat bran as a control. Finally, an in vivo study was carried out to investigate the applicability of noni meal as a feed ingredient for Holstein dairy cows. The in vitro study showed that the addition of noni meal up to 7% at 24 and 48 h of incubation did not negatively affect rumen fermentation characteristics. The in situ study showed that the rumen degradable protein content of the noni meal was high. The in vivo study showed that 1.5% noni meal can be used as a feed ingredient for Holstein dairy cows to improve the C18:1 fatty acid concentration in the milk.

**Abstract:**

In three consecutive studies, we evaluated the effects of noni *(Morinda citrifolia)* meal on rumen fermentation and degradation characteristics, production performance, physiological parameters, and milk fatty acid profile in Holstein dairy cows. In in vitro (first study) and in situ (second study) experiments, rumen fluids from two fistulated Holstein dairy cows were used. The concentration of noni meal added was 0 (control), 1, 3, 5, or 7% of the basal diet (DM basis). In the in situ experiment, wheat bran was used as a control. Triplicated bags were incubated for 0, 4, 8, 12, 24, 48, 72, or 96 h. In an in vivo experiment (third study), 38 Holstein cows (145 ± 87 days DIM; 1.8 ± 0.9 parity; 35.4 ± 6.3 kg/day milk yield) were equally assigned to the control and treatment groups (19 cows each). Basal feed and noni meal pellets (1.5% of total feed DM basis) were fed to the treatment group. The control group was also fed the basal feed and pellets containing 0% noni meal. There were no significant differences in in vitro dry matter digestibility, pH, total gas production (TGP), CH4, NH_3_-N, and volatile fatty acids (*p* > 0.05). In the in situ experiments, the crude protein (CP) rapidly soluble fraction ‘a’ (CP-a) was higher in noni meal than in wheat bran, and rumen degradable protein was also higher in noni meal than in wheat bran. In the in vivo experiments, when noni meal pellets were fed, there was no significant difference in milk yield and composition, but the triglyceride levels decreased (*p* < 0.05), the C18:1 fatty acid level increased (*p* < 0.05), and the C18:0 fatty acid level decreased (*p* < 0.05). Collectively, noni meal can be used as a feed ingredient up to 1.5% (total feed DM basis) in Holstein dairy cows and as feed supplementation to increase the C18:1 fatty acid level in milk.

## 1. Introduction

Noni (*Morinda citrifolia*) is a perennial plant belonging to the rubiaceous family and is a tropical fruit cultivated in Thailand, Indonesia, Hawaii, and Guam. It grows mainly in volcanic soils in coastal lowlands [1]. According to an Indonesian survey in 2014, 8,577,000 tons of noni were produced [2]. The main production area of noni is Indonesia. The fruit contains about 100 kinds of secondary metabolites, such as polyphenols, flavonoids, lignans, and iridoids [3,4], and has been consumed by Polynesian people for 2000 years as a traditional medicine for various diseases, being a source of, i.e., painkillers, anti-inflammatory agents, and immunostimulants [5]. The consumption of noni is likely to increase because noni contains various bioactive substances that play important roles in a human healthy life. Polyphenols have been extensively studied for their biological role in plants, antioxidant activities, health-promoting effects in ruminants [6,7,8,9,10], and the activity of rumen microbes responsible for biohydrogenation (BH) [11]. Noni meal, a by-product left after consumption of noni juice, also contains various also physiologically active substances [4]. Polyphenols have been reported to affect microbial activity [12]. Given these effects, noni meal may have various beneficiary effects on the productivity of ruminants. In addition, noni meal has high dietary fiber and protein contents [13], so it is expected that noni meal can be used as a feed ingredient for ruminants; although, the number of in vitro experiment studies in ruminants is scant; however, a previous study confirmed the reduction in total gas production and methane gas with the inclusion of noni leaves or fruits in Holstein cows [14]. Changes in blood components were confirmed when raw noni and noni pulp was fed to calves [6,9]. In the experiment in which noni was added, changes in ammonia nitrogen concentration and digestibility were confirmed [15]. In addition, an increase in VFA production was confirmed in an experiment using noni juice waste [16]. However, only one dosage was supplied (15% of total diet), while no studies have documented the use of noni meal in dairy cows.

Therefore, the purpose of this study is to explore the effect of noni meal on the rumen fermentation characteristics in an in vitro experiment and to evaluate the rumen degradation characteristics in an in situ experiment. Based on the results of in vitro and in situ experiments, this study is designed to evaluate the applicability of using noni meal at the different supplemental ratios for Holstein dairy cows as a feed ingredient supplement.

## 2. Materials and Methods

All experimental procedures involving animals were conducted in accordance with the animal testing guidelines provided by the Institutional Animal Care and Use Committee (IACUC) of Konkuk University, Seoul, Korea (KU21015, Approval number).

### 2.1. In Vitro Bach Culture

#### 2.1.1. Experimental Materials and Methods

Rumen fluid was collected from two fistulated Holstein-Friesian dairy cows 01:30 h before morning feeding. Cows were fed a total mixed ration (TMR) once a day at 08:30 h. TMR was based on 52% forage and 48% concentrated mixture. The TMR feed was dried at 65 °C for 48 h using a dry oven and then screened with a 0.5~1 mm screen. Noni meal was supplied from Indonesia and prepared in the same way as the TMR. The chemical composition of TMR was 18.87% crude protein (CP), 41.05% neutral detergent fiber (NDF), and 18.5% acid detergent fiber (ADF). The TMR used as a basal diet was placed in ANKOM bags (filter bag 58, Macedon, NY, USA) at 0.3 g each. The concentration of noni meal added was 0 (control), 1, 3, 5, or 7% of the basal diet (DM basis). Rumen fluid was filtered using a nylon filter with a pore size of 250 µm (Shanghai Bolting Cloth Manufacturing, Shanghai, China) and mixed in the same ratio. The mixed rumen fluid was transferred to the laboratory using a thermos. McDougall’s buffer [17] solution was prepared by flushing CO_2_ gas so that the pH was 6.9 at 39 °C. The buffer solution and rumen fluid were mixed in a ratio of 3:1 (*v*:*v*). After putting the ANKOM bag and noni meal into the serum bottle, 30 mL of buffer solution was added to the bottle. Argon (Ar) gas [18] was then flushed into the headspace of the serum bottle. Samples were incubated in a shaking incubator (JSSI-300C, Gongju, Korea) for 24 and 48 h at 39 °C. Bottles were repeated twice, and the experiment was repeated thrice.

#### 2.1.2. Chemical Analysis

Chemical analysis of CP, ether extract (EE), ash, and DM for the TMR and noni meal (Table 1) was performed using the Association of Official Analytical Chemists (AOAC 1990) standard method, and NDF, ADF, and ADL were analyzed using the Van Soest method [19].

#### 2.1.3. Post-Fermentation Parameters Analysis

Total gas production (TGP) was calculated from the headspace gas pressure measured using a pressure transducer (Sun Bee Instrument Inc., Seoul, Korea) [20]. The CH_4_ concentration was calculated from 0.3 mL of headspace gas sampled using a gas-tight syringe (Gastight#1001; Reno, NV, USA) and then stored in vacuum containers (Labco Exetainer, Bucks, PA, USA). The stored headspace gas was injected manually into a gas chromatograph (HP 6890 series GC system; Santa Clara, CA, USA) with a thermal conductivity detector and capillary column (HP-PLOT/Q; Santa Clara, CA, USA). The standard gas used to quantify the CH_4_ consisted of H_2_ 1.0%, CO_2_ 20.1%, CH_4_ 10.1%, and N_2_ 19.9% in He (MS Dong Min Gases, Pyeongtaek, Korea). The pH value was measured using a digital pH meter (S20 SevenEasy pH; Greifensee, Switzerland).

For volatile fatty acid (VFA) and NH_3_-N measurements, the rumen fluid samples were transferred to a 50 mL tube and stored immediately at −20 °C; the 50 mL tubes were centrifuged at 2000× *g* at 4 °C for 10 min to remove feed particles. A volume of 5 mL of supernatant of the rumen fluid was transferred to a 15 mL tube and mixed with 1 mL of HgCl_2_ 1% (*w*/*v*) solution. A 1.4 mL sample prepared for VFA analysis was transferred to a 2 mL tube and centrifuged again at 20,000× *g* at 4 °C for 20 min. Next, 1 mL of the supernatant was mixed with 25 µL of 1% (*w*/*v*) pivalic acid as an internal standard and stored in a brown vial. The VFA profile was measured using a gas chromatograph (HP 6890 series GC system; Santa Clara, CA, USA) equipped with a flame ionization detector and a capillary column (DB-FFAP; Santa Clara, CA, USA). Each sample for the VFA analysis was duplicated.

For NH_3_-N analysis, 0.5 mL of previously centrifuged sample was transferred to a 1.5 mL tube and, centrifuged again at 20,000× *g* at 4 °C for 20 min; then, 0.4 mL of the supernatant was transferred to a new 1.5 mL tube. The supernatant was used to determine the NH_3_-N concentration by a catalyzed indophenol reaction [21] using spectrophotometry (Synergy2; Winooski, VT, USA). NH_3_-N analysis was performed 3 times for each sample.

#### 2.1.4. Statistical Analysis

The results of the in vitro experiment were analyzed via a one-way ANOVA using SAS 9.4 software (SAS Institute, Cary, NC, USA). Significant differences were accepted if *p* < 0.05. A post hoc comparison was performed using Tukey’s test.

### 2.2. In Situ Experiment

#### 2.2.1. Experimental Materials and Methods

Two fistulated Holstein-Friesian cows were used to determine the rumen degradation characteristics of noni meal and wheat bran. Cows were fed a TMR once a day at 08:30 h. Wheat bran was used as a comparative control to evaluate the feed value of noni meal. All samples were dried at 65 °C for two days using a dry oven and then screened with a 0.25~1 mm screen. Each sample was placed in ANKOM bags (R1020, Macedon, NY, USA) with 7 g. The triplicated ANKOM bags were incubated in each cow of rumen for 0, 4, 8, 12, 24, 48, 72, or 96 h. Therefore, a total of [3 (noni meal, wheat bran, and Blank) × 3 (replication)] × 8 (incubation times) × 2 (animals) = 144 bags were used. To minimize the effects of inserted bags on rumen fermentation and to keep the number of inserted bags.

#### 2.2.2. Chemical Analysis

Chemical analysis of DM, CP, EE, and ash for noni meal and wheat bran (Table 2) was performed using the Association of Official Analytical Chemists (AOAC 1990) standard method, but NDF, ADF, and ADL were analyzed using the Van Soest method [19].

#### 2.2.3. Post-Incubation Procedure and Chemical Analysis

ANKOM bags were removed from the rumen, immediately washed in cold tap water, and transferred to the laboratory; then, the bags were washed under running cold tap water. They were sonicated 3 times for 5 min each using a sonicator (Power sonic 520, Hwashin Co., Gwangju, Korea). The sonicated samples were dried at 65 °C for 72 h in a dry oven for DM measurement. Crude protein was measured using the AOAC (1990) standard method. The NDF and ADF measurements were performed using the Van Soest method [19].

#### 2.2.4. Mathematical Calculation

After incubation, DM, CP, NDF, and ADF were fractionated into three parts according to their relative susceptibility. The effective rumen degradability (ERD) at rumen passage rate K_p_ (%/h) was calculated according to the following equation [22].

ERD = a + bc/(c + K_p_) (1)

In the above equation, a denotes the rapidly soluble fraction (%), b denotes the insoluble potentially degradable fraction (%), and c is a constant indicating the disappearance rate of fraction b.

RDP = a + b [c/(c + K_p_)] (2)

RUP = b [K_p_/(c + K_p_)] + [1 – (a + b)] (3)

The rumen degradable protein (RDP) and rumen undegradable protein (RUP) were calculated according to the equations above (NRC, 2001).

### 2.3. In Vivo Experiment

#### 2.3.1. Animals and Diets

Thirty-eight Holstein cows (145 ± 87 days DIM; 1.8 ± 0.9 parity; 35.4 ± 6.3 kg/day milk yield; 5.42 ± 0.89% milk fat; 3.07 ± 0.18% milk protein; 4.71 ± 0.14% milk lactose) were used and equally assigned to the control and treatment groups in this study (19 cows each). The experimental period was 6 weeks. Water was provided *ad libitum* throughout the study.

During the experiment, all cows were fed a basal diet consisting of a TMR (52% forage and 48% concentrate) with an extra amount of concentrate (2 kg) and tall fescue hay (2.37 kg). The control group was fed a basal diet and 3 kg/d of pellets without noni meal. The treatment group was fed a basal diet and 3 kg/d of pellets with 15% noni meal. The chemical composition and energy value of the control pellets and the noni meal pellets were similar. Only the noni meal pellet contained 15% noni meal, which was the highest possible level of noni meal that can be used to meet the similar chemical compositions in both groups. Thus, the concentration of noni meal was 1.5% of the total feed (DM basis). The in vitro experiments showed no negative effect on rumen fermentation following the use of noni meal up to 7%, but in in vivo study, it was fed in the form of pellets to prevent the selection of dietary components and improve the palatability of the feed. The maximum concentration of noni meal in the pellet that could make a difference in nutrients from the control pellet was 15%. The chemical compositions of all diets used during all experimental periods were as follows and they were provided according to NRC nutrient requirements (2001; Table 3). Feed was offered once daily at 10:00 h, and pellets with 15% noni meal (DM basis) and pellets without noni meal were supplemented as topdressing on the TMR.

#### 2.3.2. Feed Sampling and Analysis

Samples of TMR, concentrates, tall fescue, and pellets were collected at 0, 21, and 42 d. All samples were dried at 65 °C for 48 h using a drying oven for chemical analysis. The chemical analysis of DM, CP, EE, and ash was performed using the AOAC (1990) method, and NDF, ADF, and ADL were analyzed using the Van Soest method [19].

#### 2.3.3. Milk Sampling and Analysis

Cows were milked twice daily at 08:00 and 18:00 h, and milk yield was recorded at each milking. Milk samples were collected at 0, 21, and 42 days using automatic samplers in the morning and evening of the sampling day. For milk component analysis, milk samples were pooled and transferred into 50 mL tubes with 2-bromo-2nitropropane-1,3 diol (Broad Spectrum Microtabs II, Norwood, MA, USA) and then stored at 4 °C until analysis. Milk components were analyzed using MilkoScan FT1 (Hillerod, Denmark), and samples were analyzed for protein, fat, lactose, milk urea nitrogen (MUN), solid-not-fat (SNF), somatic cells, acetone, and beta-hydroxybutyrate (BHB). The milk fat yield, milk protein yield, energy-corrected milk (ECM), and 3.5% fat-corrected milk (FCM) were calculated using the equations provided by NRC nutrient requirements (2001).

For milk fatty acid analysis, pooled milk samples were transferred to a new 50 mL tube. Milk fatty acids were analyzed via gas chromatography/FID (7890B GC System, Agilent Technologies, Santa Clara, CA, USA). FAME Mix standard (Sigma, St. Louis, MO, USA) was the standard used to determine the fatty acid content and fatty acid ratio.

#### 2.3.4. Blood Sampling and Analysis

Blood samples were collected from the jugular vein and were immediately collected into ethylene diamine-tetra-acetic acid (EDTA)-treated vacutainers (Franklin Lakes, NJ, USA) and serum tubes (Plymouth, UK) at approximately 2 h before feeding on days 0, 21, and 42. Whole blood was analyzed for white blood cells (WBC), lymphocytes, monocytes, granulocytes, red blood cell numbers (RBC), hemoglobin, hematocrit, platelets, mean corpuscular volume (MCV), mean corpuscular hemoglobin concentration (MCHC), mean platelet volume (MPV), mean corpuscular hemoglobin (MCH), platelet distribution width (PDWc), plateletcrit (PCT), red cell distribution width (RDWc), percent granulocytes, percent lymphocytes, and percent monocytes using a hematology analyzer (VetScan HM2, Abaxis, Union City, CA, USA).

Blood serum was separated by centrifugation (2200 rpm for 15 min at 4 °C) and stored at −80 °C. The samples of serum were analyzed for glutamic-oxaloacetic transaminase (GOT), glutamic pyruvic transaminase (GPT), blood urea nitrogen (BUN), triglyceride (TG), total cholesterol (TCHO), and glucose (GLC) using a chemical analyzer (HITACHI Automatic Analyzer Model 7180, Gyeonggi-do, Korea).

#### 2.3.5. Statistical Analysis

All data were analyzed using PROC MIXED of SAS 9.4 software (SAS Institute, Cary, NC, USA). Milk yield, milk composition, blood cells, and serum metabolites were analyzed using repeated measures analysis. Covariance structures were chosen among UN, ANTE (1), CS, CSH, VC, UNR, and TOEP based on the smallest value of the Akaike information criterion (AIC). Differences were declared statistically significant at *p* < 0.05 and statistical tendencies at 0.05 ≤ *p* < 0.10.

## 3. Results

### 3.1. In Vitro Experiment

#### Effects of Noni Meal on Rumen Fermentation Characteristics

We analyzed the IVDMD, TGP, CH_4_, NH_3_-N, and VFA contents of rumen fluid after adding various levels of noni meal for 24 h or 48 h (Table 4 and Table 5). As the concentration of noni meal increased, TGP showed a tendency (*p* < 0.10) to increase and then decrease at 48 h. However, the TGP was not lower than that of the control when noni meal was added up to 7%. When noni meal was added from 0% to 7%, there was no significant difference (*p* > 0.05) in all parameters.

### 3.2. In Situ Experiment

#### Degradation Characteristics

The rumen degradation parameters and effective rumen degradability (ERD) of the noni meal and wheat bran are shown in Table 6.

As expected, dry matter rapidly soluble fraction ‘a’ (DM-a), dry matter insoluble potentially degradable fraction ‘b’ (DM-b), and DM ERD of noni meal were lower compared to wheat bran.

The crude protein (CP) rapidly soluble fraction ‘a’ (CP-a) and rumen degradable protein (RDP) of noni meal were higher compared to wheat bran.

The neutral detergent fiber (NDF) rapidly soluble fraction ‘a’ (NDF-a) of noni meal was higher compared to wheat bran. The NDF insoluble potentially degradable fraction ‘b’ (NDF-b) and NDF ERD of noni meal were lower compared to wheat bran.

The acid detergent fiber (ADF) rapidly soluble fraction ‘a’ (ADF-a), ADF insoluble potentially degradable fraction ‘b’ (ADF-b), and ADF ERD of noni meal were lower compared to wheat bran.

### 3.3. In Vivo Experiment

#### 3.3.1. Milk Production and Characteristics

The effects of the noni meal pellets on milk production are shown in Table 7. The milk yield, milk protein, milk true protein, milk fat, milk lactose, milk urea nitrogen, solid-not-fat, beta-hydroxybutyrate, milk protein yield, milk true protein yield, milk fat yield, energy-corrected milk, and 3.5% fat-corrected milk were analyzed, but there was no significant difference in these parameters between the control group and the treatment group (*p* > 0.05).

#### 3.3.2. Complete Blood Cell Counts

The effects of the noni meal pellets on complete blood cell count (CBC) are shown in Table 8. There were no significant differences (*p* > 0.05) between the control and treatment groups regarding WBCs, lymphocytes, monocytes, granulocytes, RBCs, Hemoglobin, hematocrit, platelet, MCV, MCHC, MPV, MCH, RDWC, PCT, PDWc, and monocyte percent. Granulocyte percent showed a tendency to increase (*p* < 0.10) in the treatment group as compared to the control group. Lymphocyte percent showed a tendency to decrease (*p* < 0.10) in the treatment group as compared to the control group.

#### 3.3.3. Serum Metabolites

Serum metabolite results are shown in Table 9. There were no significant differences (*p* > 0.05) between the control and treatment groups in terms of GOT, GPT, BUN, TCHO, and GLC. TG was significantly decreased (*p* < 0.05) in the treatment group compared to the control group.

#### 3.3.4. Milk Fatty Acid Compositions

The results of the analysis of milk fatty acids are shown in Table 10. The C18:0 fatty acid level was significantly decreased (*p* < 0.05) in the treatment group compared to the control group. The C18:1 fatty acid content was significantly increased (*p* < 0.05) in the treatment group compared to the control group. C20:5n3 fatty acid showed a tendency to increase in the treatment group compared to the control group (*p* < 0.10).

## 4. Discussion

### 4.1. In Vitro Experiment

The results of the in vitro experiment were consistent with the results of Anjani et al. [2]. In their study, there was no difference in pH, total VFA, and NH_3_-N when 5% seedless noni waste was substituted into feed, but there was a difference when 10% was substituted. In this study, the ADF content of the noni meal was 47.92% of DM, and the lignin content was 14.45% of DM, which is high (Table 1). It is known that high ADF and lignin content are reasons for the decrease in DM digestibility [23]. However, the addition of up to 7% noni meal did not affect the DM digestibility, and these results are consistent with the study by Anjani et al. [2]. According to a previous study by Barraza-Elenes et al. [24], which analyzed the physiologically active substances of noni bagasse (with or without seeds) obtained through the juice extraction process from noni fruits, the tannin contents of 0, 1, 3, 5, and 7% noni meal are expected to be 0, 20.43, 61.29, 102.15, and 143.01 µg CE/g DM, respectively. Previously, Javanegara et al. [25] reported a CH_4_ reduction effect by tannin when the concentration of tannin ranged from 0 to 250 mg CE/g DM, which is 1700 times higher than the tannin concentration measured in the noni meal in this study. Consistently, supplementation of 7% did not reduce CH_4_, which could be explained by the remarkably small amount of tannin in the noni meal. In this experiment, when noni was added up to 5%, TGP increased, but it showed a tendency (*p* < 0.10) to decrease at 7%. In contrast, Paengkoum et al. [26] reported a decrease in cumulative gas production by supplementing with 2–6% DM of Mangosteen-Peel enriched tannins. Additionally, Vieira and Borba [27] suggested that supplementation with quebracho tannins at 2.5% and 5% DM suppressed cumulative gas production. This may be due to the tannin concentration. Therefore, if the noni meal concentration is increased to more than 7%, TGP may be reduced.

### 4.2. In Situ Experiment

In this experiment, wheat bran was used as a control to confirm the rumen degradation characteristics of noni meal. According to a previous study [28], when K_p_ 3.27%/h was used, the DM ERD of wheat bran was 68.9%/h, similar to the results of the present study. In another previous study [29], when K_p_ 3%/h was used, the DM ERD of wheat bran was 74.9%/h because the experiment was conducted for up to 120 h. In this study, when K_p_ 4%/h was used to evaluate the rumen degradation characteristics of noni meal and wheat bran, the DM ERD values were 53.46 and 62.88%/h, respectively, and the DM ERD values of wheat bran showed similar results to those of the previous study [28,29]. This may be due to the similar chemical compositions of noni meal and wheat bran. The chemical composition (DM basis) of the noni meal was 12.18% CP, 47.92% NDF, and 37.72% ADF. The chemical composition (DM basis) of the wheat bran was 15.22% CP, 39.63% NDF, and 12.06% ADF.

Habib et al. [30] reported that when K_p_ 2%/h was used, the CP ERD of wheat bran was 85.1%/h. In this experiment, when K_p_ 2%/h was used, the CP ERD was calculated as 84.55%/h, confirming the accuracy of the rumen degradation characteristics obtained in this experiment. The CP-a of noni meal was 71.27%, which means that most of the protein in noni meal is composed of NH_3_, NO_3_, and amino acid peptides, which are immediately degraded in the rumen. According to a previous study [31], an increase in the concentration of ammonia in the rumen causes intraruminal toxicity. However, there was no significant difference (*p* > 0.05) in our in vitro study results regarding in ammonia production (Table 4 and Table 5), even when noni meal was supplemented up to 7%, and we thus assumed that there was no rumen toxicity. Therefore, noni meal can be used as a ruminant feed ingredient for increasing RDP.

In a previous study [29], with respect to the NDF ERD, a negative correlation with the ADL content was reported, so the NDF ERD of noni meal was lower than that of wheat bran. In addition, another study [23] revealed that high lignin content could lower feed digestibility. However, the results of the in vitro study (Table 4 and Table 5) showed that even when noni meal was supplemented up to 7%, there was no significant difference (*p* > 0.05) in IVDMD.

### 4.3. In Vivo Experiment

The absence of any significant difference (*p* > 0.05) in the milk production results (Table 7) from the in vivo experiment might be attributable to the results of the in vitro experiment (Table 4 and Table 5). There was no difference (*p* > 0.05) in VFA production when noni meal was added up to 7% in vitro. Therefore, supplementation of noni meal up to 7% may not negatively affect milk production in vivo. Noni contains proxeronine, which regulates the structure and function of proteins [32], and has the effect of killing *Escherichia coli*, which is associated with mastitis [7]. Therefore, it was expected that the somatic cell count would decrease when noni meal pellets were fed, but there was no significant difference in this study (*p* > 0.05). These results may be due to the use of healthy cows in both the control and treatment groups. In agreement with the results obtained in this study when animals were fed noni meal at 450 g/animal, in a previous study [8] no change in milk yield was observed when animals were fed noni juice at 100 mL/animal. The total polyphenol content of noni was reported to be twice that of noni meal [24]. The total polyphenol content of the noni meal fed in this in vivo experiment was 116.2 mg/kg DM. Previous studies [33,34] that supplied total polyphenols at a lower concentration than that used in this in vivo experiment showed the same result, with no change in milk yield. However, milk yield was increased when chestnut tannin extract at a higher concentration than that used in this in vivo experiment was fed [35]. Therefore, it may be that there was no change in milk yield due to the low concentration of the noni meal.

A previous study [9] reported a positive effect of significantly reducing WBCs by feeding noni pulp, and there were no significant differences in lymphocytes, red blood cells, hemoglobin, and hematocrit. Therefore, in this in vivo study, it was expected that WBCs would decrease when noni meal pellets were fed, but there was no difference. However, the same results were obtained for lymphocytes, red blood cells, hemoglobin, and hematocrit in a previous study [9] and in this in vivo experiment. These results may be explained by a difference in the concentrations of noni used in this study and in the previous study [9], resulting in no decrease in the white blood cell concentration. Through a previous study [24], the total flavonoids in the noni meal fed in this study were calculated as 180 mg/kg body weight (BW). Similar to our experimental results, in a previous study [34], when 100 mg/kg BW of alfalfa flavonoid extract was supplemented, the lymphocyte percentage decreased and the neutrophil granulocyte percentage increased. Granulocytes play an important role in innate immunity, and lymphocytes play an important role in acquired immunity. The increasing trend of the granulocyte percentage indicates that the animal’s ability to resist infection can be improved [34]. Harizi et al. [36] reported that flavonoids can inhibit lymphocyte activation and proliferation. Although the lymphocyte percentage showed a tendency to decrease in the treatment group as compared to the control group, it was within the normal range (>48%) [37]. Therefore, the flavonoids in noni meal may affect the innate immunity of Holstein dairy cows.

According to a previous study [6], when raw noni was supplemented, TCHO, TG, GLU, and BUN were significantly reduced (*p* < 0.05) in the treatment group. Noni juice contains higher levels of bioactive substances than noni meal [24]. Hence, raw noni will also contain higher levels of bioactive substances than noni meal. The previous study [6] showed opposite results that may be attributed to feeding three times more per BW than we did in this in vivo study. Triglycerides were significantly lower in the treatment group compared to the control group. A previous study [38] reported that triglycerides in the blood, and omega-3 fatty acids, reduce fat deposition in adipose tissue by inhibiting adipogenic enzymes and increasing β-oxidation. In addition, it is documented that omega-6 fatty acids increase membrane permeability and, thus, increase the intracellular triglyceride content [39]. Therefore, fatty acid composition analysis of the pellets of the control group and the pellets of the treatment group was performed. There was no difference between omega-3 and omega-6 fatty acids, suggesting that changes in blood triglycerides were not due to differences in diet. According to a previous study [40], a triglycerides concentration of 0.12 mmol/L or less can be identified as an indicator of ketosis. The concentration of triglycerides was 0.356 mmol/L in the control group and 0.265 mmol/L in the treatment group, so it was confirmed that the values in this study were within the normal range.

Vasta et al. and Aguiar et al. [33,41] reported that phenolic compounds affect fermentation in the rumen. Therefore, the increase in C18:1 fatty acid and decrease in C18:0 fatty acid may be due to phenolic compounds that may have inhibited *Butyrivibrio proteoclasticus* and reduced bio-hydrogenation in the treatment group. Long-chain omega-3 fatty acids are converted from ALA to eicosapentaenoic acid (EPA; C20:5n3) through desaturase elongation and desaturase elongation processes such as ∆5-desaturase and ∆6-desaturase [42]. Gilani et al. [43] reported that tannin (polyphenol) regulates ∆9-desaturase. Noni meal contains tannins and other polyphenols, which may regulate other desaturases (∆5-desaturase and ∆6-desaturase) that induce desaturation. Therefore, 20:5n3 fatty acid may have tended to increase (*p* < 0.10) in the treatment group as compared to the control group. Aguiar et al. [33] and Purba et al. [12] reported an increase in C18:1 fatty acid and a decrease in C18:0 fatty acid when supplementing with phenolic compounds. Their results were consistent with the results of this study. In contrast, when phenolic compounds were supplemented in previous studies [12,33], MUFA increased, while SFA decreased. This may be due to the high concentration of phenolic compounds supplied, resulting in the desaturation of several fatty acids. In contrast, CLA was increased when supplemented with phenolic compounds. In previous studies [12,33], a fat source (linoleic acid) was added together, or this may be due to a different form of the phenolic compound supplied.

## 5. Conclusions

These experimental results showed that there was no deleterious effect on rumen fermentation when noni meal was added up to 7%. Moreover, noni meal can be a good source of RDP. When noni meal pellets were fed, the triglyceride levels significantly decreased, the C18:1 fatty acid level significantly increased, and the C18:0 fatty acid level decreased. In conclusion, up to 1.5% (DM basis) noni meal can be used as a feed inclusion in the diets of Holstein dairy cows. In addition, if further research is conducted, it is suggested that noni meal can be supplemented as a functional feed additive for Holstein dairy cows to improve their milk quality, such as by increasing the C18:1 fatty acid level.

## Figures and Tables

**Table 1:** Chemical compositions of diets.

Items ^1^	TMR	Noni Meal
Dry matter (%)	66.70	90.87
Chemical composition (DM %)		
CP	18.87	12.18
EE	4.06	3.09
NDF	41.05	47.92
ADF	18.50	37.72
ADL	1.72	14.45
Ash	8.93	7.56

^1^ CP, crude protein; EE, ether extract; NDF, neutral detergent fiber; ADF, acid detergent fiber; ADL, acid detergent insoluble lignin.

**Table 2:** Chemical compositions of diets.

Items ^1^	Noni Meal	Wheat Bran
Dry matter (%)	90.87	89.65
Chemical composition (DM %)		
CP	12.18	15.22
EE	3.09	4.30
NDF	47.92	39.63
ADF	37.72	12.06
ADL	14.45	1.82
Ash	7.56	4.67

^1^ CP, crude protein; EE, ether extract; NDF, neutral detergent fiber; ADF, acid detergent fiber; ADL, acid detergent insoluble lignin.

**Table 3:** Chemical compositions of diets.

	TMR	Concentrate	Tall Fescue	Noni Meal Pellet	Control Pellet
Analyzed values ^1^ (DM %)					
CP	17.79	19.41	10.66	20.46	21.32
EE	5.16	4.98	1.59	3.94	4.06
NDF	41.01	30.25	62.71	31.50	30.67
NDIP	10.07	13.04	6.94	11.63	13.03
NDFn	30.94	17.21	55.77	19.87	17.64
ADF	20.86	12.29	32.29	12.77	12.09
ADIP	4.63	6.80	1.73	5.53	4.79
ADL	4.42	2.47	3.63	1.75	2.10
Ash	8.77	9.14	9.06	7.57	7.53
Estimated values ^2^					
NFC	27.27	36.21	15.98	36.53	36.42
TDN1_x_	67.89	76.88	59.25	77.85	78.52
NE_L_	1.48	1.74	1.33	1.76	1.78

^1^ CP, crude protein; EE, ether extract; NDF, neutral detergent fiber; NDIP, neutral detergent insoluble crude protein; NDFn, NDF—NDICP; ADF, acid detergent fiber; ADIP, acid detergent insoluble crude protein; ADL acid detergent insoluble lignin. ^2^ NFC, non-fiber carbohydrate; TDN, total digestible nutrient; NE_L_, net energy for lactation; The estimated values were calculated according to the NRC (2001).

**Table 4:** Effect of noni meal on rumen in vitro dry matter digestibility (IVDMD), pH, total gas production (TGP), CH_4_, and NH_3_-N production, and volatile fatty acids (VFAs) profiles in 24 h incubation.

	Noni Meal Supplementation (% of DM of Diet)					SEM	*p*-Value
Items ^1^	0	1	3	5	7		
IVDMD, g/dg	77.93	78.68	77.29	77.99	76.37	1.057	0.47
pH	6.38	6.40	6.38	6.36	6.38	0.065	0.56
TGP, mL/g DM degraded	246.32	248.03	256.41	256.56	256.07	11.736	0.29
CH_4_, mL/g DM degraded	45.39	46.53	46.93	46.57	47.57	0.794	0.42
NH_3_-N, mg/dL	77.06	69.73	83.57	78.85	77.74	10.634	0.88
Total VFA, mM	56.96	56.34	56.92	58.23	57.81	2.469	0.97
Acetate, mmol/100 mol	59.53	60.37	60.60	60.14	61.09	1.461	0.21
Propionate, mmol/100 mol	21.83	21.95	21.85	21.93	21.69	1.403	0.60
iso-butyrate, mmol/100 mol	1.06	1.02	1.06	1.03	1.02	0.066	0.77
Butyrate, mmol/100 mol	14.28	13.94	13.39	14.13	13.89	0.349	0.17
iso-valerate, mmol/100 mol	1.04	0.99	1.00	1.02	0.98	0.042	0.75
Valerate, mmol/100 mol	1.81	1.74	1.72	1.75	1.69	0.080	0.48
BCFA, mmol/100 mol	2.10	2.01	2.03	2.05	2.00	0.103	0.78
A:P ratio	2.71	2.80	2.80	2.77	2.80	0.050	0.36

^1^ IVDMD, in vitro dry matter digestibility; TGP, total gas production; BCFA, branched-chain fatty acid; A:P ratio, acetate and propionate ratio.

**Table 5:** Effect of noni meal on rumen in vitro dry matter digestibility (IVDMD), pH, total gas production (TGP), CH_4_, and NH_3_-N production, and volatile fatty acids (VFAs) profiles in 48 h incubation.

	Noni Meal Supplementation (% of DM of Diet)					SEM	*p*-Value
Items ^1^	0	1	3	5	7		
IVDMD, g/dg	82.82	81.86	82.97	82.16	83.53	1.071	0.53
pH	6.33	6.32	6.31	6.31	6.28	0.064	0.18
TGP, mL/g DM degraded	270.06	275.53	278.97	279.78	274.54	8.441	0.06
CH_4_, mL/g DM degraded	55.08	55.29	55.92	57.09	54.87	1.390	0.28
NH_3_-N, mg/dL	134.82	137.40	122.83	120.04	127.02	12.113	0.55
Total VFA, mM	65.14	67.74	75.16	76.96	80.43	7.588	0.31
Acetate, mmol/100 mol	60.33	60.57	60.83	61.40	61.40	1.726	0.50
Propionate, mmol/100 mol	21.49	21.33	21.68	21.34	21.60	1.544	0.92
iso-butyrate, mmol/100 mol	1.40	1.39	1.35	1.36	1.32	0.068	0.45
Butyrate, mmol/100 mol	13.29	13.38	13.13	13.13	12.96	0.351	0.77
iso-valerate, mmol/100 mol	1.40	1.40	1.36	1.36	1.32	0.075	0.76
Valerate, mmol/100 mol	1.95	1.93	1.74	1.55	1.68	0.124	0.12
BCFA, mmol/100 mol	2.80	2.79	2.71	2.72	2.64	0.142	0.63
A:P ratio	2.83	2.88	2.86	2.89	2.89	0.091	0.91

^1^ IVDMD, in vitro dry matter digestibility; TGP, total gas production; BCFA, branched-chain fatty acid; A:P ratio, acetate and propionate ratio.

**Table 6:** Rumen in situ dry matter (DM), crude protein (CP), neutral detergent fiber (NDF) and acid detergent fiber (ADF) degradation parameters and effective rumen degradability of noni meal and wheat bran.

Items	Noni Meal	Wheat Bran
DM degradation parameters ^1^		
a	38.09	41.62
b	22.94	44.46
c	0.08	0.04
DM effective rumen degradability ^2^ (%/h)	53.46	62.88
CP degradation parameters		
a	71.27	46.84
b	23.00	48.82
c	0.09	0.07
Rumen degradable proteins (% of CP/h) ^3^	87.38	77.56
Rumen undegradable proteins (% of CP/h) ^3^	12.62	22.44
NDF degradation parameters		
a	3.62	0.43
b	24.06	70.21
c	0.06	0.03
NDF effective rumen degradability	18.45	31.91
ADF degradation parameters		
a	2.24	7.56
b	19.77	43.77
c	0.08	0.03
ADF effective rumen degradability	15.54	25.92

^1^ a = rapidly soluble fraction (%); b = insoluble potentially degradable fraction (%); c = constant for disappearance rate of b fraction. ^2^ Effective rumen degradability (ERD) was calculated by the equation from Orkov and McDonald (1979); ERD = a + bc/(c + K_p_), where K_p_ was the rumen passage rate assumed to be 4%/h. ^3^ Rumen degradable proteins (RDP) and rumen undegradable proteins (RUP) were calculated by the equation from NRC (2001); RDP = a + b[c/(c + K_p_)], RUP = [1 − (a + b) + b[K_p_/(c + K_p_)]].

**Table 7:** Effects of noni meal on milk yield and composition in Holstein dairy cows.

Items ^3^	Control ^1^	Treatment ^2^	SEM	*p*-Value
Milk yield, kg/day	34.37	34.74	0.814	0.848
Milk protein, %	3.12	3.14	0.033	0.623
Milk true protein, %	2.61	2.63	0.028	0.667
Milk fat, %	5.01	5.35	0.135	0.277
Lactose, %	4.70	4.68	0.025	0.656
Milk urea nitrogen, mg/dL	16.30	16.08	0.367	0.676
Solid-not-fat, %	8.45	8.45	0.044	0.992
Acetone, mM	0.02	0.02	0.004	0.875
BHB, mM	0.06	0.07	0.005	0.339
SCC, 10^3^	85.39	126.79	15.352	0.213
Milk protein yield, kg/d	1.07	1.08	0.024	0.835
Milk true protein yield, kg/d	0.89	0.91	0.019	0.901
Milk fat yield, kg/d	1.75	1.85	0.072	0.550
ECM, kg/d	41.62	43.13	1.283	0.621
3.5% FCM, kg/d	43.25	45.04	1.439	0.598

^1^ Control = Control pellet. ^2^ Treatment = Noni meal pellet (contains 15% noni meal). ^3^ BHB, beta-hydroxybutyrate; SCC, somatic cell count; ECM, energy corrected milk; FCM, fat corrected milk.

**Table 8:** Effects of noni meal on complete blood cell counts in Holstein dairy cows.

Items ^3^	Control ^1^	Treatment ^2^	SEM	*p*-Value
WBCs, 10^9^/L	12.14	11.32	0.412	0.387
Lymphocytes, 10^9^/L	7.76	6.41	0.400	0.163
Monocytes, 10^9^/L	0.60	0.67	0.068	0.504
Granulocytes, 10^9^/L	3.78	4.25	0.160	0.112
RBCs, 10^12^/L	7.14	7.17	0.109	0.892
Hemoglobin, g/dL	11.25	11.25	0.153	0.996
Hematocrit, %	31.79	31.95	0.385	0.851
Platelet, 10^9^/L	387.05	375.67	12.225	0.550
MCV, fL	44.81	44.81	0.462	1.000
MCHC, g/dL	35.38	35.20	0.136	0.497
MPV, fL	7.69	7.45	0.078	0.101
MCH, pg	15.82	15.75	0.151	0.965
PDWc, %	33.87	33.40	0.273	0.345
PCT, %	0.30	0.28	0.009	0.315
RDWc, %	20.20	22.98	2.126	0.359
GR, %	32.93	38.23	1.357	0.069
LY, %	61.65	55.80	1.651	0.091
MO, %	5.42	5.81	0.563	0.675

^1^ Control = Control pellet. ^2^ Treatment = Noni meal pellet (contains 15% noni meal). ^3^ WBC, white blood cell; RBC, red blood cell; MCV, mean corpuscular volume; MCHC, mean corpuscular hemoglobin concentration; MPV, mean platelet volume; MCH, mean corpuscular hemoglobin; PDWc, platelet distribution width; PCT, plateletcrit; RDWc, red cell distribution width; GR, granulocyte percent; LY, lymphocyte percent; MO, monocyte percent.

**Table 9:** Effects of noni meal on serum metabolites in Holstein dairy cows.

Items ^3^	Control ^1^	Treatment ^2^	SEM	*p*-Value
GOT, U/L^3^	80.79	87.26	1.996	0.160
GPT, U/L^3^	37.40	35.47	0.647	0.164
BUN, mg/dL	19.49	18.76	0.347	0.324
TCHO, mg/dL	316.98	304.39	7.387	0.464
TG, mg/dL	6.42	4.77	0.411	0.035
GLC, mg/dL	54.70	55.21	1.255	0.691

^1^ Control = Control pellet. ^2^ Treatment = Noni meal pellet (contains 15% noni meal). ^3^ GOT, glutamic-oxaloacetic transaminase; GPT, glutamic pyruvic transaminase; BUN, blood urea nitrogen; TCHO, total cholesterol; TG, triglycerides; GLC, glucose.

**Table 10:** Effects of noni meal on milk fatty acids composition (%) in Holstein dairy cows.

Items ^3^	Control ^1^	Treatment ^2^	SEM	*p*-Value
C16:0	30.185	30.699	0.351	0.524
C18:1n9c	21.084	19.889	0.311	0.116
C18:1 (TVA)	0.878	1.196	0.077	0.003
C18:0	12.583	11.596	0.641	0.038
CLA	0.486	0.495	0.020	0.783
C18:2n6t	0.265	0.267	0.012	0.913
C18:2n6c	2.999	2.963	0.104	0.816
C18:3n6	0.034	0.036	0.001	0.578
C20:3n6	0.151	0.142	0.004	0.212
C20:4n6	0.194	0.190	0.006	0.625
C18:3n3	0.184	0.186	0.004	0.853
C20:3n3	0.031	0.033	0.001	0.478
C20:5n3	0.026	0.028	0.001	0.069
C22:6n3	0.012	0.009	0.002	0.104
SFA	71.141	71.882	0.759	0.403
MUFA	24.430	23.728	0.821	0.412
PUFA	4.429	4.391	0.103	0.852
ω6	3.643	3.598	0.109	0.764
ω3	0.253	0.256	0.006	0.857
ω6/ω3	14.399	14.055	0.184	0.623

^1^ Control = Control pellet. ^2^ Treatment = Noni meal pellet (contains 15% noni meal). ^3^ Items = TVA, trans11 vaccenic acid; CLA, conjugated linoleic acid; SFA, saturated fatty acid; MUFA, mono-unsaturated fatty acid; PUFA, poly-unsaturated fatty acid.

## Data Availability

The data can be provided upon a reasonable request and to the requested person only.
